# Efficient Actor-Critic Algorithm with Hierarchical Model Learning and Planning

**DOI:** 10.1155/2016/4824072

**Published:** 2016-10-03

**Authors:** Shan Zhong, Quan Liu, QiMing Fu

**Affiliations:** ^1^School of Computer Science and Technology, Soochow University, Suzhou, Jiangsu 215000, China; ^2^School of Computer Science and Engineering, Changshu Institute of Technology, Changshu, Jiangsu 215500, China; ^3^Collaborative Innovation Center of Novel Software Technology and Industrialization, Jiangsu 210000, China; ^4^Key Laboratory of Symbolic Computation and Knowledge Engineering of Ministry of Education, Jilin University, Changchun 130012, China; ^5^College of Electronic & Information Engineering, Suzhou University of Science and Technology, Jiangsu, Suzhou 215000, China

## Abstract

To improve the convergence rate and the sample efficiency, two efficient learning methods AC-HMLP and RAC-HMLP (AC-HMLP with *ℓ*_2_-regularization) are proposed by combining actor-critic algorithm with hierarchical model learning and planning. The hierarchical models consisting of the local and the global models, which are learned at the same time during learning of the value function and the policy, are approximated by local linear regression (LLR) and linear function approximation (LFA), respectively. Both the local model and the global model are applied to generate samples for planning; the former is used only if the state-prediction error does not surpass the threshold at each time step, while the latter is utilized at the end of each episode. The purpose of taking both models is to improve the sample efficiency and accelerate the convergence rate of the whole algorithm through fully utilizing the local and global information. Experimentally, AC-HMLP and RAC-HMLP are compared with three representative algorithms on two Reinforcement Learning (RL) benchmark problems. The results demonstrate that they perform best in terms of convergence rate and sample efficiency.

## 1. Introduction and Related Work

Reinforcement Learning (RL) [1–4], a framework for solving the Markov Decision Process (MDP) problem, targets generating the optimal policy by maximizing the expected accumulated rewards. The agent interacts with its environment and receives information about the current state at each time step. After the agent chooses an action according to the policy, the environment will transition to a new state while emitting a reward. RL can be divided into two classes, online and offline. Online method learns by interacting with the environment, which easily incurs the inefficient use of data and the stability issue. Offline or batch RL [5] as a subfield of dynamic programming (DP) [6, 7] can avoid the stability issue and achieve high sample efficiency.

DP aims at solving optimal control problems, but it is implemented backward in time, making it offline and computationally expensive for complex or real-time problems. To avoid the curse of dimensionality in DP, approximate dynamic programming (ADP) received much attention to obtain approximate solutions of the Hamilton-Jacobi-Bellman (HJB) equation by combining DP, RL, and function approximation [8]. Werbos [9] introduced an approach for ADP which was also called adaptive critic designs (ACDs). ACDs consist of two neural networks (NNs), one for approximating the critic and the other for approximating the actor, so that DP can be solved approximately forward in time. Several synonyms about ADP and ACDs mainly include approximate dynamic programming, asymptotic dynamic programming, heuristic dynamic programming, and neurodynamic programming [10, 11].

The iterative nature of the ADP formulation makes it natural to design the optimal discrete-time controllers. Al-Tamimi et al. [12] established a heuristic dynamic programming algorithm based on value iteration, where the convergence is proved in the context of general nonlinear discrete systems. Dierks et al. [13] solved the optimal control of nonlinear discrete-time systems by using two processes, online system identification and offline optimal control training, without the requirement of partial knowledge about the system dynamics. Wang et al. [14] focused on applying iterative ADP algorithm with error boundary to obtain the optimal control law, in which the NNs are adopted to approximate the performance index function, compute the optimal control policy, and model the nonlinear system.

Extensions of ADP for continuous-time systems face the challenges involved in proving stability and convergence meanwhile ensuring the algorithm being online and model-free. To approximate the value function and improve the policy for continuous-time system, Doya [15] derived a temporal difference (TD) error-based algorithm in the framework of HJB. Under a measure of input quadratic performance, Murray et al. [16] developed a stepwise ADP algorithm in the context of HJB. Hanselmann et al. [17] put forward a continuous-time ADP formulation, where Newton's method is used in the second-order actor adaption to achieve the convergence of the critic. Recently, Bhasin et al. [18] built an actor-critic-identifier (ACI), an architecture that represents the actor, critic, and model by taking NNs as nonlinearly parameterized approximators while the parameters of NNs are updated by least-square method.

All the aforementioned ADP variants utilized the NN as the function approximator; however, linear parameterized approximators are usually more preferred in RL, because they make it easier to understand and analyze the theoretical properties of the resulting RL algorithms [19]. Moreover, most of the above works did not learn a model online to accelerate the convergence rate and improve the sample efficiency. Actor-critic (AC) algorithm was introduced in [20] for the first time; many variants which approximated the value function and the policy by linear function approximation have been widely used in continuous-time systems since then [21–23]. By combining model learning and AC, Grondman et al. [24] proposed an improved learning method called Model Learning Actor-Critic (MLAC) which approximates the value function, the policy, and the process model by LLR. In MLAC, the gradient of the next state with respect to the current action is computed for updating the policy gradient, with the goal of improving the convergence rate of the whole algorithm. In their latter work [25], LFA takes the place of LLR as the approximation method for value function, the policy, and the process model. Enormous samples are still required when only using such a process model to update the policy gradient. Afterward, Costa et al. [26] derived an AC algorithm by introducing Dyna structure called Dyna-MLAC which approximated the value function, the policy, and the model by LLR as MLAC did. The difference is that Dyna-MLAC applies the model not only in updating the policy gradient but also in planning [27]. Though planning can improve the sample efficiency to a large extent, the model learned by LLR is just a local model so that the global information of samples is yet neglected.

Though the above works learn a model during learning of the value function and the policy, only the local information of the samples is utilized. If the global information of the samples can be utilized reasonably, the convergence performance will be improved further. Inspired by this idea, we establish two novel AC algorithms called AC-HMLP and RAC-HMLP (AC-HMLP with *ℓ*_2_-regularization). AC-HMLP and RAC-HMLP consist of two models, the global model and the local model. Both models incorporate the state transition function and the reward function for planning. The global model is approximated by LFA while the local model is represented by LLR. The local and the global models are learned simultaneously at each time step. The local model is used for planning only if the error does not surpass the threshold, while the global planning process is started at the end of an episode, so that the local and the global information can be kept and utilized uniformly.

The main contributions of our work on AC-HMLP and RAC-HMLP are as follows:

1. Develop two novel AC algorithms based on hierarchal models. Distinguishing from the previous works, AC-HMLP and RAC-HMLP learn a global model, where the reward function and the state transition function are approximated by LFA. Meanwhile, unlike the existing model learning methods [28–30] which represent a feature-based model, we directly establish a state-based model to avoid the error brought by inaccurate features.
2. As MLAC and Dyna-MLAC did, AC-HMLP and RAC-HMLP also learn a local model by LLR. The difference is that we design a useful error threshold to decide whether to start the local planning process. At each time step, the real-next state is computed according to the system dynamics whereas the predicted-next state is obtained from LLR. The error between them is defined as the state-prediction error. If this error does not surpass the error threshold, the local planning process is started.
3. The local model and the global model are used for planning uniformly. The local and the global models produce local and global samples to update the same value function and the policy; as a result the number of the real samples will decrease dramatically.
4. Experimentally, the convergence performance and the sample efficiency are thoroughly analyzed. The sample efficiency which is defined as the number of samples for convergence is analyzed. RAC-HMLP and AC-HMLP are also compared with S-AC, MLAC, and Dyna-MLAC in convergence performance and sample efficiency. The results demonstrate that RAC-HMLP performs best and AC-HMLP performs second best, and both of them outperform the other three methods.

This paper is organized as follows: Section 2 reviews some background knowledge concerning MDP and the AC algorithm.  Section 3 describes the hierarchical model learning and planning.  Section 4 specifies our algorithms—AC-HMLP and RAC-HMLP. The empirical results of the comparisons with the other three representative algorithms are analyzed in Section 5.  Section 6 concludes our work and then presents the possible future work.

## 2. Preliminaries

### 2.1. MDP

RL can solve the problem modeled by MDP. MDP can be represented as four-tuple (*X*, *U*, *ρ*, *f*):

(1) *X* is the state space. *x*_*t*_ ∈ *X* denotes the state of the agent at time step *t*.
(2) *U* represents the action space. *u*_*t*_ ∈ *U* is the action which the agent takes at the time step *t*.
(3) *ρ* : *X* × *U* → *ℝ* denotes the reward function. At the time step *t*, the agent locates at a state *x*_*t*_ and takes an action *u*_*t*_ resulting in next state *x*_*t*+1_ while receiving a reward *r*_*t*_ = *ρ*(*x*_*t*_, *u*_*t*_).
(4) *f* : *X* × *U* → *X* is defined as the transition function. *f*(*x*_*t*_, *u*_*t*_, *x*_*t*+1_) is the probability of reaching the next state *x*_*t*+1_ after executing *u*_*t*_ at the state *x*_*t*_.

Policy *h* : *X* → *U* is the mapping from the state space *X* to the action space *U*, where the mathematical set of *h* depends on specific domains. The goal of the agent is to find the optimal policy *h* ^*∗*^ that can maximize the cumulative rewards. The cumulative rewards are the sum or discounted sum of the received rewards and here we use the latter case.

Under the policy *h*, the value function *V* ^*h*^ : *X* → *ℝ* denotes the expected cumulative rewards, which is shown as

(1) $$\begin{array}{c} V^{h}\left(\;x\;\right)=E_{h}\left\{\;\overset{\infty }{\underset{k=0}{\sum }}\gamma^{k}r_{t+k+1}\;|\;x=x_{t}\;\right\}, \end{array}$$

where *γ* ∈ [0,1] represents the discount factor. *x*_*t*_ is the current state.

The optimal state-value function *V* ^*∗*^(*x*) is computed as

(2) $$\begin{array}{c} V^{*}\left(\;x\;\right)=\underset{h}{\max}\;V\left(\;x\;\right),\;\forall x\in X. \end{array}$$

Therefore, the optimal policy *h* ^*∗*^ at state *x* can be obtained by

(3) $$\begin{array}{c} h^{*}\left(\;x\;\right)=\arg\underset{h}{\max}{\;V}^{h}\left(\;x\;\right),\;\forall x\in X. \end{array}$$

### 2.2. AC Algorithm

AC algorithm mainly contains two parts, actor and critic, which are stored separately. Actor and critic are also called the policy and value function, respectively. The actor-only methods approximate the policy and then update its parameter along the direction of performance improving, with the possible drawback being large variance resulting from policy estimation. The critic-only methods estimate the value function by approximating a solution to the Bellman equation; the optimal policy is found by maximizing the value function. Other than the actor-only methods, the critic-only methods do not try to search the optimal policy in policy space. They just estimate the critic for evaluating the performance of the actor; as a result the near-optimality of the resulting policy cannot be guaranteed. By combining the merits of the actor and the critic, AC algorithms were proposed where the value function is approximated to update the policy.

The value function and the policy are parameterized by *V*(*x*, *θ*) and *h*(*x*, *β*), where *θ* and *β* are the parameters of the value function and the policy, respectively. At each time step *t*, the parameter *θ* is updated as

(4) $$\begin{array}{c} \theta_{t+1}=\theta_{t}+\alpha_{c}\delta_{t}\frac{\partial V\left(x,\theta \right)}{\partial \theta }\;x=x_{t},\;\;\theta =\theta_{t}, \end{array}$$

where

(5) $$\begin{array}{c} \delta_{t}=r_{t}+\gamma V\left(\;x_{t},\theta_{t}\;\right)-V\left(\;x_{t},\theta_{t}\;\right), \end{array}$$

denoting the TD-error of the value function. ∂*V*(*x*, *θ*)/∂*θ* represents the feature of the value function. The parameter *α*_*c*_ ∈ [0,1] is the learning rate of the value function.

Eligibility is a trick to improve the convergence via assigning the credits to the previously visited states. At each time step *t*, the eligibility can be represented as

(6) $$\begin{array}{c} e_{t}\left(\;x\;\right)=\left\{\begin{array}{cc} \frac{\partial V\left(x,\theta \right)}{\partial \theta } & x_{t}=x\\ \lambda \gamma e_{t-1}\left(x\right) & x_{t}\neq x, \end{array}\right. \end{array}$$

where *λ* ∈ [0,1] denotes the trace-decay rate.

By introducing the eligibility, the update for *θ* in (4) can be transformed as

(7) $$\begin{array}{c} \theta_{t+1}=\theta_{t}+\alpha_{c}\delta_{t}e_{t}\left(\;x\;\right). \end{array}$$

The policy parameter *β*_*t*_ can be updated by

(8) $$\begin{array}{c} \beta_{t+1}=\beta_{t}+\alpha_{a}\delta_{t}\Delta u_{t}\frac{\partial h\left(x,\beta \right)}{\partial \beta }, \end{array}$$

where ∂*h*(*x*, *β*)/∂*β* is the feature of the policy. Δ*u*_*t*_ is a random exploration term conforming to zero-mean normal distribution. *α*_*a*_ ∈ [0,1] is the learning rate of the policy.

S-AC (Standard AC algorithm) serves as a baseline to compare with our method, which is shown in Algorithm 1. The value function and the policy are approximated linearly in Algorithm 1, where TD is used as the learning algorithm.

## 3. Hierarchical Model Learning and Planning 

### 3.1. Why to Use Hierarchical Model Learning and Planning

The model in RL refers to the state transition function and the reward function. When the model is established, we can use any model-based RL method to find the optimal policy, for example, DP. Model-based methods can significantly decrease the number of the required samples and improve the convergence performance. Inspired by this idea, we introduce the hierarchical model learning into AC algorithm so as to make it become more sample-efficient. Establishing a relative accurate model for the continuous state and action spaces is still an open issue.

The preexisting works are mainly aimed at the problems with continuous states but discrete actions. They approximated the transition function in the form of probability matrix which specifies the transition probability from the current feature to the next feature. The indirectly observed features result in the inaccurate feature-based model. The convergence rate will be slowed significantly by using such an inaccurate model for planning, especially at each time step in the initial phase.

To solve these problems, we will approximate a state-based model instead of the inaccurate feature-based model. Moreover, we will introduce an additional global model for planning. The global model is applied only at the end of each episode so that the global information can be utilized as much as possible. Using such a global model without others will lead to the loss of valuable local information. Thus, like Dyna-MLAC, we also approximate a local model by LLR and use it for planning at each time step. The difference is that a useful error threshold is designed for the local planning in our method. If the state-prediction error between the real-next state and the predicted one does not surpass the error threshold, the local planning process will be started at the current time step. Therefore the convergence rate and the sample efficiency can be improved dramatically by combining the local and global model learning and planning.

### 3.2. Learning and Planning of the Global Model

The global model establishes separate equations for the reward function and the state transition function of every state component by linear function approximation. Assume the agent is at state *x*_*t*_ = {*x*_*t*,1_,…, *x*_*t*,*K*_}, where *K* is the dimensionality of the state *x*_*t*_; the action *u*_*t*_ is selected according to the policy *h*; then all the components {*x*_*t*+1,1_′, *x*_*t*+1,2_′,…, *x*_*t*+1,*K*_′} of the next state *x*_*t*+1_′ can be predicted as

(9) $$\begin{array}{c} x_{t+1,1}^{\prime }={\eta_{t,1}}^{T}\phi \left(\;x_{t,1},x_{t,2},\ldots ,x_{t,K},u_{t}\;\right)\\ x_{t+1,2}^{\prime }={\eta_{t,2}}^{T}\phi \left(\;x_{t,1},x_{t,2},\ldots ,x_{t,K},u_{t}\;\right)\\ \vdots \\ x_{t+1,K}^{\prime }={\eta_{t,K}}^{T}\phi \left(\;x_{t,1},x_{t,2},\ldots ,x_{t,K},u_{t}\;\right), \end{array}$$

where *η*_*t*,*i*_ = (*η*_*t*,*i*1_, *η*_*t*,*i*2_,…,*η*_*t*,*iD*_)^T^, 1 ≤ *i* ≤ *K*, is the parameter of the state transition function corresponding to the *i*th component of the current state at time step *t*, with *D* being the dimensionality of the feature *ϕ*(*x*_*t*,1_, *x*_*t*,2_,…, *x*_*t*,*K*_, *u*_*t*_).

Likewise, the reward *r*_*t*+1_′ can be predicted as

(10) $$\begin{array}{c} r_{t+1}^{\prime }={\varsigma_{t}}^{T}\phi \left(\;x_{t,1},x_{t,2},\ldots ,x_{t,K},u_{t}\;\right), \end{array}$$

where *ς*_*t*_ = (*ς*_*t*1_, *ς*_*t*2_,…, *ς*_*tD*_) is the parameter of the reward function at time step *t*.

After *η*_*t*,1_, *η*_*t*,2_,…, *η*_*t*,*K*_ and *ς* are updated, the model can be applied to generate samples. Let the current state be *x*_*t*_ = (*x*_*t*,1_, *x*_*t*,2_,…, *x*_*t*,*k*_); after the action *u*_*t*_ is executed, the parameters *η*_*t*+1,*i*_  (1 ≤ *i* ≤ *K*) can be estimated by the gradient descent method, shown as

(11) ηt+1,1=ηt,1+αmxt+1,1−xt+1,1′ϕxt,1,xt,2,…,xt,K,utηt+1,2=ηt,2+αmxt+1,2−xt+1,2′ϕxt,1,xt,2,…,xt,K,ut⋮ηt+1,K=ηt,K+αmxt+1,K−xt+1,K′ϕxt,1,xt,2,…,xt,K,ut,

where *α*_*m*_ ∈ [0,1] is the learning rate of the model. *x*_*t*+1_ is the real-next state obtained according to the system dynamics, where *x*_*t*+1,*i*_  (1 ≤ *i* ≤ *K*) is its *i*th component. (*x*_*t*+1,1_′, *x*_*t*+1,2_′,…, *x*_*t*+1,*K*_′) is the predicted-next state according to (9).

The parameter *ς*_*t*+1_ is estimated as

(12) $$\begin{array}{c} \varsigma_{t+1}=\varsigma_{t}+\alpha_{m}\left(\;r_{t+1}-r_{t+1}^{\prime }\;\right)\phi \left(\;x_{t,1},x_{t,2},\ldots ,x_{t,K},u_{t}\;\right), \end{array}$$

where *r*_*t*+1_ is the real reward reflected by the system dynamics while *r*_*t*+1_′ is the predicted reward obtained according to (10).

### 3.3. Learning and Planning of the Local Model

Though the local model also approximates the state transition function and the reward function as the global model does, LLR is served as the function approximator instead of LFA. In the local model, a memory storing the samples in the form of (*x*_*t*_, *u*_*t*_, *x*_*t*+1_, *r*_*t*+1_) is maintained. At each time step, a new sample is generated from the interaction and it will take the place of the oldest one in the memory. Not all but only* L*-nearest samples in the memory will be selected for computing the parameter matrix Γ ∈ *ℝ* ^(*K*+1)×(*K*+2)^ of the local model. Before achieving this, the input matrix *X* ∈ *ℝ* ^(*K*+2)×*L*^ and the output matrix *Y* ∈ *ℝ* ^(*K*+1)×*L*^ should be prepared as follows:

(13) X=x1,1x2,1⋯xL,1x1,2x2,2⋯xL,2⋮⋮⋮⋮x1,Kx2,K⋯xL,Ku1u2⋯uL11⋯1,Y=x2,1x3,1⋯xL+1,1x2,2x3,2⋯xL+1,2⋮⋮⋮⋮x2,Kx3,K⋯xL+1,Kr2r3⋯rL+1.

The last row of *X* consisting of ones is to add a bias on the output. Every column in the former *K* + 1 lines of *X* corresponds to a state-action pair; for example, the *i*th column is the state-action (*x*_*i*_, *u*_*i*_)^T^, 1 ≤ *i* ≤ *L*. *Y* is composed of *L* next states and rewards corresponding to *X*.

Γ can be obtained via solving *Y* = Γ*X* as

(14) $$\begin{array}{c} \Gamma =YX^{T}{\left(\;XX^{T}\;\right)}^{-1}. \end{array}$$

Let the current input vector be [*x*_*t*,1_,…, *x*_*t*,*K*_, *u*_*t*_, 1]^T^; the output vector [*x*_*t*+1,1_′,…, *x*_*t*+1,*K*_′, *r*_*t*+1_′]^T^ can be predicted by

(15) $$\begin{array}{c} {\left[\;x_{t+1,1}^{\prime },\ldots ,x_{t+1,k}^{\prime },r_{t+1}^{\prime }\;\right]}^{T}=\Gamma {\left[\;x_{t,1},\ldots ,x_{t,k},u_{t},1\;\right]}^{T}, \end{array}$$

where [*x*_*t*,1_,…, *x*_*t*,*K*_] and [*x*_*t*+1,1_′,…, *x*_*t*+1,*K*_′] are the current state and the predicted-next state, respectively. *r*_*t*+1_′ is the predicted reward.

Γ is estimated according to (14) at each time step; thereafter the predicted-next state and the predicted reward can be obtained by (15). Moreover, we design an error threshold to decide whether local planning is required. We compute the state-prediction error between the real-next state and the predicted-next state at each time step. If this error does not surpass the error threshold, the local planning process will be launched. The state-prediction error is formulated as

(16) Ert=max⁡xt+1,1′−xt+1,1xt+1,1,xt+1,2′−xt+1,2xt+1,2,…,xt+1,K′−xt+1,Kxt+1,K,rt+1′−rt+1rt+1.

Let the error threshold be *ξ*; then the local model will be used for planning only if *Er*_*t*_ ≤ *ξ* at time step *t*. In the local planning process, a sequence of locally simulated samples in the form of (*x*_*t*,1_,…, *x*_*t*,*K*_, *u*_*t*_, *x*_*t*+1,1_′,…, *x*_*t*+1,*K*_′, *r*′) are generated to improve the convergence of the same value function and the policy as the global planning process does.

## 4. Algorithm Specification

### 4.1. AC-HMLP

AC-HMLP algorithm consists of a main algorithm and two subalgorithms. The main algorithm is the learning algorithm (see Algorithm 2), whereas the two subalgorithms are local model planning procedure (see Algorithm 3) and global model planning procedure (see Algorithm 4), respectively. At each time step, the main algorithm learns the value function (see line (26) in Algorithm 2), the policy (see line (27) in Algorithm 2), the local model (see line (19) in Algorithm 2), and the global model (see lines (10)~(11) in Algorithm 2).

There are several parameters which are required to be determined in the three algorithms. *r* and *λ* are discount factor and trace-decay rate, respectively. *α*_*a*_, *α*_*c*_, and *α*_*m*_ are the corresponding learning rates of the value function, the policy, and the global model. *M*_size is the capacity of the memory. *ξ* denotes the error threshold. *L* determines the number of selected samples which is used to fit the LLR. *σ* ^2^ is the variance that determines the region of the exploration. *P*_*l*_ and *P*_*g*_ are the planning times for local model and global model. Some of these parameters have empirical values, for example, *r* and *λ*. The others have to be determined by observing the empirical results.

Notice that Algorithm 3 starts planning at the state *x*_*t*_ which is passed from the current state in Algorithm 2, while Algorithm 4 uses *x*_0_ as the initial state. The reason for using different initializations is that the local model is learned according to the* L*-nearest samples of the current state *x*_*t*_, whereas the global model is learned from all the samples. Thus, it is reasonable and natural to start the local and global planning process at the states *x*_*t*_ and *x*_0_, respectively.

### 4.2. AC-HMLP with *ℓ*_2_-Regularization

Regression approaches in machine learning are generally represented as minimization of a square loss term and a regularization term. The *ℓ*_2_-regularization also called ridge regress is a widely used regularization method in statistics and machine learning, which can effectively prohibit overfitting of learning. Therefore, we introduce *ℓ*_2_-regularization to AC-HMLP in the learning of the value function, the policy, and the model. We term this new algorithm as RAC-HMLP.

The goal of learning the value function is to minimize the square of the TD-error, which is shown as

(17) minθ⁡∑t=0numberrt+1+γVxt+1′,θt−Vxt,θt+lcθt2,

where number represents the number of the samples. *ℓ*_*c*_ ≥ 0 is the regularization parameter of the critic. ‖*θ*_*t*_‖^2^ is the *ℓ*_2_-regularization which penalizes the growth of the parameter vector *θ*_*t*_, so that the overfitting to noise samples can be avoided.

The update for the parameter *θ* of the value function in RAC-HMLP is represented as

(18) $$\begin{array}{c} \theta_{t+1}=\theta_{t}\left(\;1-\frac{\alpha_{c}}{number}l_{c}\;\right)+\frac{\alpha_{c}}{number}\overset{number}{\underset{s=0}{\sum }}e_{s}\left(\;x\;\right)\delta_{s}. \end{array}$$

The update for the parameter *β* of the policy is shown as

(19) βt+1=βt1−αanumberla+αanumber∑s=0numberϕxsδsΔus,

where *ℓ*_*a*_ ≥ 0 is the regularization parameter of the actor.

The update for the global model can be denoted as

(20) ηt+1,1=ηt,11−αmnumberlm+αmnumber·∑s=1numberϕxs,1,…,xs,K,usxs+1,1−xs+1,1′⋮ηt+1,K=ηt,K1−αmnumberlm+αmnumber·∑s=1numberϕxs,1,…,xs,K,usxs+1,K−xs+1,1′

(21) ςt+1=ςt1−αmnumberlm+αmnumber·∑s=1numberϕxs,1,…,xs,K,usrs+1−rs+1′,

where *ℓ*_*m*_ ≥ 0 is the regularization parameter for the model, namely, the state transition function and the reward function.

After we replace the update equations of the parameters in Algorithms 2, 3, and 4 with (18), (19), (20), and (21), we will get the resultant algorithm, RAC-HMLP. Except for the above update equations, the other parts of RAC-HMLP are the same with AC-HMLP, so we will not specify here.

## 5. Empirical Results and Analysis

AC-HMLP and RAC-HMLP are compared with S-AC, MLAC, and Dyna-MLAC on two continuous state and action spaces problems, pole balancing problem [31] and continuous maze problem [32].

### 5.1. Pole Balancing Problem

**Figure 1:**
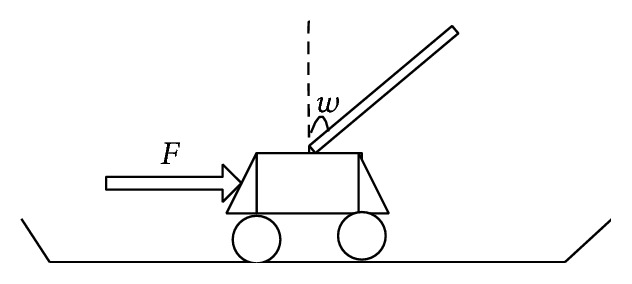
Pole balancing problem.

Pole balancing problem is a low-dimension but challenging benchmark problem widely used in RL literature, shown in Figure 1.

There is a car moving along the track with a hinged pole on its top. The goal is to find a policy which can guide the force to keep the pole balance. The system dynamics is modeled by the following equation:

(22) $$\begin{array}{c} \ddot{w}=\frac{g\sin\left(w\right)-vml\dot{w}\sin\left(2w\right)/2-v\cos\left(w\right)F}{4l/3-vml\cos^{2}\left(w\right)}, \end{array}$$

where *w* is the angle of the pole with the vertical line. $\dot{w}$ and $\ddot{w}$ are the angular velocity and the angular acceleration of the pole. *F* is the force exerted on the cart. The negative value means the force to the right and otherwise means to the left. *g* is the gravity constant with the value *g* = 9.81 m/s^2^. *m* and *l* are the length and the mass of the pole, which are set to *m* = 2.0 kg and *l* = 0.5 m, respectively. *v* is a constant with the value 1/(*m* + *m*_*c*_), where *m*_*c*_ = 8.0 kg is the mass of the car.

The state $x=(w,\dot{w})$ is composed of *w* and $\dot{w}$ which are bounded by [−*π*, *π*] and [−2, 2], respectively. The action is the force *F* bounded by [−50 N, 50 N]. The maximal episode is 300, and the maximal time step for each episode is 3000. If the angle of the pole with the vertical line is not exceeding *π*/4 at each time step, the agent will receive a reward 1; otherwise the pole will fall down and receive a reward −1. There is also a random noise applying on the force bounded by [−10 N, 10 N]. An episode ends when the pole has kept balance for 3000 time steps or the pole falls down. The pole will fall down if |*w* | ≥ *π*/4. To approximate the value function and the reward function, the state-based feature is coded by radial basis functions (RBFs), shown as

(23) $$\begin{array}{c} \phi_{i}\left(\;x\;\right)=e^{-\left(1/2\right){\left(x-c_{i}\right)}^{T}B^{-1}\left(x-c_{i}\right)}, \end{array}$$

where *c*_*i*_ denotes the *i*th center point locating over the grid points {−0.6, −0.4, −0.2,0, 0.2,0.4,0.6}×{−2,0, 2}. *B* is a diagonal matrix containing the widths of the RBFs with *σ*_*w*_^2^ = 0.2 and $\sigma_{\dot{w}}^{2}=2$. The dimensionality *z* of *ϕ*(*x*) is 21. Notice that the approximations of the value function and the policy only require the state-based feature. However, the approximation of the model requires the state-action-based feature. Let *x* be $\left(w,\dot{w},u\right)$; we can also compute its feature by (23). In this case, *c*_*i*_ locates over the points {−0.6, −0.4, −0.2,0, 0.2,0.4,0.6}×{−2,0, 2}×{−50, −25,0, 25,50}. The diagonal elements of *B* are *σ*_*w*_^2^ = 0.2, $\sigma_{\dot{w}}^{2}=2$, and *σ*_*F*_^2^ = 10. The dimensionality *z* of $\phi (w,\dot{w},u)$ is 105. Either the state-based or the state-action-based feature has to be normalized so as to make its value be smaller than 1, shown as

(24) $$\begin{array}{c} \overset{-}{\phi_{i}}\left(\;x\;\right)=\frac{\phi_{i}\left(x\right)}{\sum_{i=1}^{z}\phi_{i}\left(x\right)}, \end{array}$$

where *z* > 0 is the dimensionality of the feature.

RAC-HMLP and AC-HMLP are compared with S-AC, MLAC, and Dyna-MLAC on this experiment. The parameters of S-AC, MLAC, and Dyna-MLAC are set according to the values mentioned in their papers. The parameters of AC-HMLP and RAC-HMLP are set as shown in Table 1.

**Figure 2:**
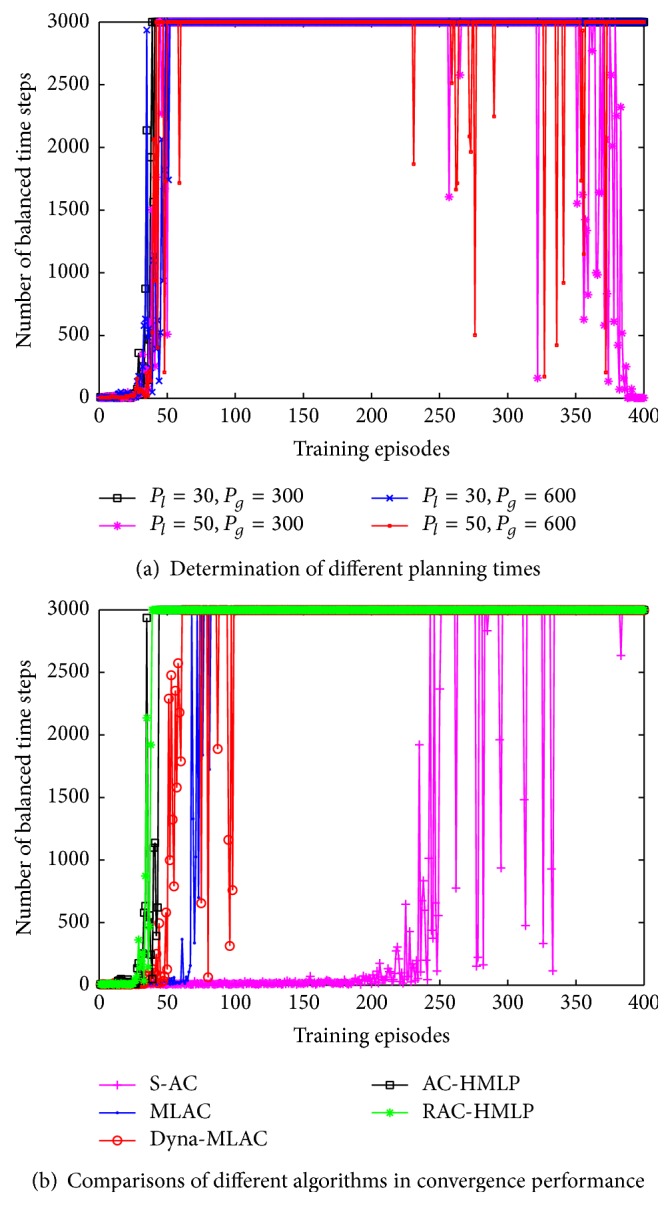
Comparisons of different planning times and different algorithms.

The planning times may have significant effect on the convergence performance. Therefore, we have to determine the local planning times *P*_*l*_ and the global planning times *P*_*g*_ at first. The selective settings for the two parameters are (30, 300), (50, 300), (30, 600), and (50, 600). From Figure 2(a), it is easy to find out that (*P*_*l*_ = 30, *P*_*g*_ = 300) behaves best in these four settings, with 41 episodes for convergence. (*P*_*l*_ = 30, *P*_*g*_ = 600) learns fastest in the early 29 episodes, but it converges until the 52nd episode. (*P*_*l*_ = 30, *P*_*g*_ = 600) and (*P*_*l*_ = 30, *P*_*g*_ = 300) have identical local planning times but different global planning times. Generally, the more the planning times, the faster the convergence rate, but (*P*_*l*_ = 30, *P*_*g*_ = 300) performs better instead. The global model is not accurate enough at the initial time. Planning through such an inaccurate global model will lead to an unstable performance. Notice that (*P*_*l*_ = 50, *P*_*g*_ = 300) and (*P*_*l*_ = 50, *P*_*g*_ = 600) behave poorer than (*P*_*l*_ = 30, *P*_*g*_ = 600) and (*P*_*l*_ = 30, *P*_*g*_ = 300), which demonstrates that planning too much via the local model will not perform better. (*P*_*l*_ = 50, *P*_*g*_ = 300) seems to converge at the 51st episode but its learning curve fluctuates heavily after 365 episodes, not converging any more until the end. Like (*P*_*l*_ = 50, *P*_*g*_ = 300), (*P*_*l*_ = 50, *P*_*g*_ = 600) also has heavy fluctuation but converges at the 373rd episode. Evidently, (*P*_*l*_ = 50, *P*_*g*_ = 300) performs slightly better than (*P*_*l*_ = 50, *P*_*g*_ = 300). Planning through the global model might solve the nonstability problem caused by planning of the local model.

The convergence performances of the five methods, RAC-HMLP, AC-HMLP, S-AC, MLAC, and Dyna-MLAC, are shown in Figure 2(b). It is evident that our methods RAC-HMLP and AC-HMLP have the best convergence performances. RAC-HMLP and AC-HMLP converge at the 39th and 41st episodes. Both of them learn quickly in the primary phase, but the learning curve of RAC-HMLP seems to be steeper than that of AC-HMLP. Dyna-MLAC learns faster than MLAC and S-AC in the former 74 episodes, but it converges until the 99th episode. Though MLAC behaves poorer than Dyna-MLAC, it requires just 82 episodes to converge. The method with the slowest learning rate is S-AC where the pole can keep balance for 3000 time steps for the first time at the 251st episode. Unfortunately, it converges until the 333rd episode. RAC-HMLP converges fastest which might be caused by introducing the *ℓ*_2_-regularization. Because the *ℓ*_2_-regularization does not admit the parameter to grow rapidly, the overfitting of the learning process can be avoided effectively. Dyna-MLAC converges faster than MLAC in the early 74 episodes but its performance is not stable enough embodied in the heavy fluctuation from the 75th to 99th episode. If the approximate-local model is distinguished largely from the real-local model, then planning through such an inaccurate local model might lead to an unstable performance. S-AC as the only method without model learning behaves poorest among the five. These results show that the convergence performance can be improved largely by introducing model learning.

**Figure 3:**
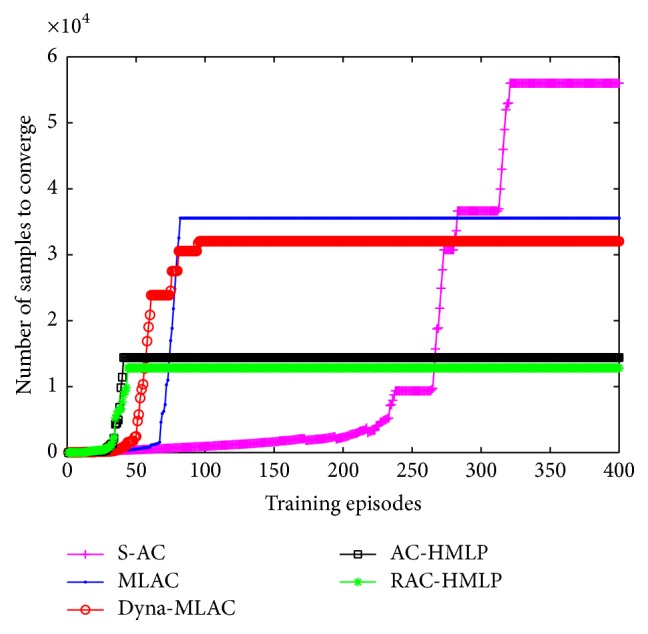
Comparisons of sample efficiency.

The comparisons of the five different algorithms in sample efficiency are shown in Figure 3. It is clear that the numbers of the required samples for S-AC, MLAC, Dyna-MLAC, AC-HMLP, and RAC-HMLP to converge are 56003, 35535, 32043, 14426, and 12830, respectively. Evidently, RAC-HMLP converges fastest and behaves stably all the time, thus requiring the least samples. Though AC-HMLP requires more samples to converge than RAC-HMLP, it still requires far fewer samples than the other three methods. Because S-AC does not utilize the model, it requires the most samples among the five. Unlike MLAC, Dyna-MLAC applies the model not only in updating the policy but also in planning, resulting in a fewer requirement for the samples.

**Figure 4:**
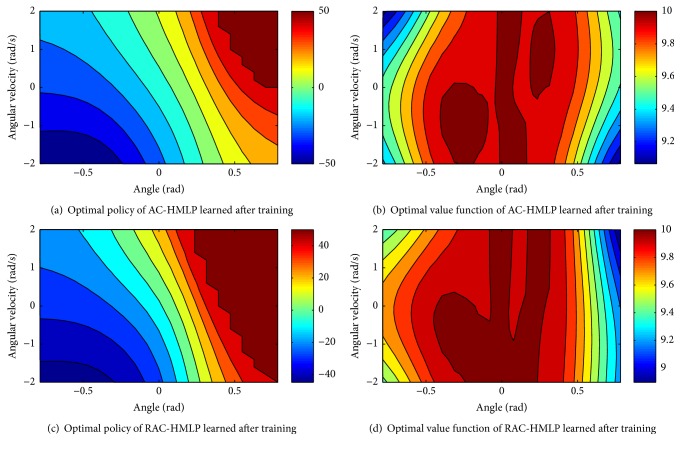
Optimal policy and value function learned by AC-HMLP and RAC-HMLP.

The optimal policy and optimal value function learned by AC-HMLP after the training ends are shown in Figures 4(a) and 4(b), respectively, while the ones learned by RAC-HMLP are shown in Figures 4(c) and 4(d). Evidently, the optimal policy and the optimal value function learned by AC-HMLP and RAC-HMLP are quite similar, but RAC-HMLP seems to have more fine-grained optimal policy and value function.

As for the optimal policy (see Figures 4(a) and 4(c)), the force becomes smaller and smaller from the two sides (left side and right side) to the middle. The top-right is the region requiring a force close to 50 N, where the direction of the angle is the same with that of angular velocity. The values of the angle and the angular velocity are nearing the maximum values *π*/4 and 2. Therefore, the largest force to the left is required so as to guarantee that the angle between the upright line and the pole is no bigger than *π*/4. It is opposite in the bottom-left region where a force attributing to [−50 N, −40 N] is required to keep the angle from surpassing −*π*/4. The pole can keep balance with a gentle force close to 0 in the middle region. The direction of the angle is different from that of angular velocity in the top-left and bottom-right regions; thus a force with the absolute value which is relatively large but smaller than 50 N is required.

In terms of the optimal value function (see Figures 4(b) and 4(d)), the value function reaches a maximum in the region satisfying −2 ≤ *w* ≤ 2. The pole is more prone to keep balance even without applying any force in this region, resulting in the relatively larger value function. The pole is more and more difficult to keep balance from the middle to the two sides with the value function also decaying gradually. The fine-grained value of the left side compared to the right one might be caused by the more frequent visitation to the left side. More visitation will lead to a more accurate estimation about the value function.

**Figure 5:**
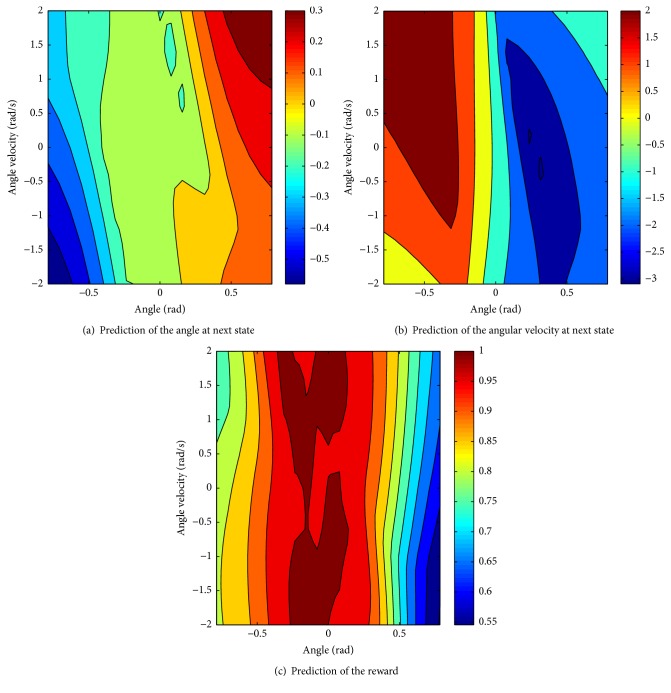
Prediction of the next state and reward according to the global model.

After the training ends, the prediction of the next state and the reward for every state-action pair can be obtained through the learned model. The predictions for the next angle *w*, the next angular velocity $\dot{w}$, and the reward for any possible state are shown in Figures 5(a), 5(b), and 5(c). It is noticeable that the predicted-next state is always near the current state in Figures 5(a) and 5(b). The received reward is always larger than 0 in Figure 5(c), which illustrates that the pole can always keep balance under the optimal policy.

### 5.2. Continuous Maze Problem

**Figure 6:**
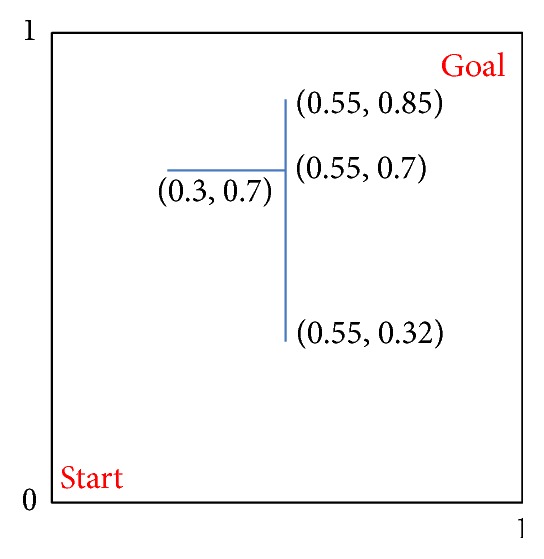
Continuous maze problem.

Continuous maze problem is shown in Figure 6, where the blue lines with coordinates represent the barriers. The state (*x*, *y*) consists of the horizontal coordinate *x* ∈ [0,1] and vertical coordinate *y* ∈ [0,1]. Starting from the position “Start,” the goal of the agent is to reach the position “Goal” that satisfies *x* + *y* > 1.8. The action is to choose an angle bounded by [−*π*, *π*] as the new direction and then walk along this direction with a step 0.1. The agent will receive a reward −1 at each time step. The agent will be punished with a reward −400 multiplying the distance if the distance between its position and the barrier exceeds 0.1. The parameters are set the same as the former problem, except for the planning times. The local and global planning times are determined as 10 and 50 in the same way of the former experiment. The state-based feature is computed according to (23) and (24) with the dimensionality *z* = 16. The center point *c*_*i*_ locates over the grid points {0.2,0.4,0.6,0.8}×{0.2,0.4,0.6,0.8}. The diagonal elements of *B* are *σ*_*x*_^2^ = 0.2 and *σ*_*y*_^2^ = 0.2. The state-action-based feature is also computed according to (23) and (24). The center point *c*_*i*_ locates over the grid points {0.2,0.4,0.6,0.8}×{0.2,0.4,0.6,0.8}×{−3, −1.5,0, 1.5,3} with the dimensionality *z* = 80. The diagonal elements of *B* are *σ*_*x*_^2^ = 0.2, *σ*_*y*_^2^ = 0.2, and *σ*_*u*_^2^ = 1.5.

**Figure 7:**
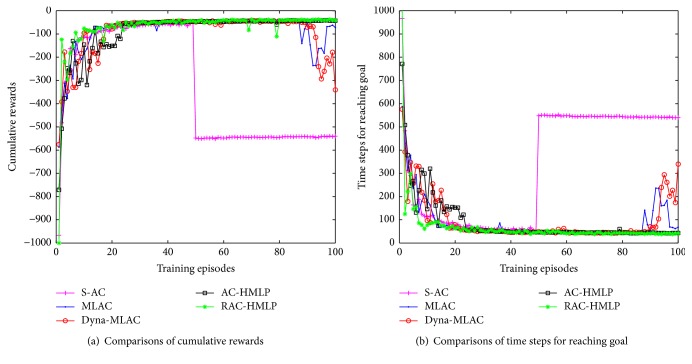
Comparisons of cumulative rewards and time steps for reaching goal.

RAC-HMLP and AC-HMLP are compared with S-AC, MLAC, and Dyna-MLAC in cumulative rewards. The results are shown in Figure 7(a). RAC-HMLP learns fastest in the early 15 episodes, MLAC behaves second best, and AC-HMLP performs poorest. RAC-HMLP tends to converge at the 24th episode, but it really converges until the 39th episode. AC-HMLP behaves steadily starting from the 23rd episode to the end and it converges at the 43rd episode. Like the former experiment, RAC-HMLP performs slightly better than AC-HMLP embodied in the cumulative rewards −44 compared to −46. At the 53rd episode, the cumulative rewards of S-AC fall to about −545 quickly without any ascending thereafter. Though MLAC and Dyna-MLAC perform well in the primary phase, their curves start to descend at the 88th episode and the 93rd episode, respectively. MLAC and Dyna-MLAC learn the local model to update the policy gradient, resulting in a fast learning rate in the primary phase. However, they do not behave stably enough near the end of the training. Too much visitation to the barrier region might cause the fast descending of the cumulative rewards.

The comparisons of the time steps for reaching goal are simulated, which are shown in Figure 7(b). Obviously, RAC-HMLP and AC-HMLP perform better than the other three methods. RAC-HMLP converges at 39th episode while AC-HMLP converges at 43rd episode, with the time steps for reaching goal being 45 and 47. It is clear that RAC-HMLP still performs slightly better than AC-HMLP. The time steps for reaching goal are 548, 69, and 201 for S-AC, MLAC, and Dyna-MLAC. Among the five methods, RAC-HMLP not only converges fastest but also has the best solution, 45. The poorest performance of S-AC illustrates that model learning can definitely improve the convergence performance. As in the former experiment, Dyna-MLAC behaves poorer than MLAC during training. If the model is inaccurate, planning via such model might influence the estimations of the value function and the policy, thus leading to a poor performance in Dyna-MLAC.

**Figure 8:**
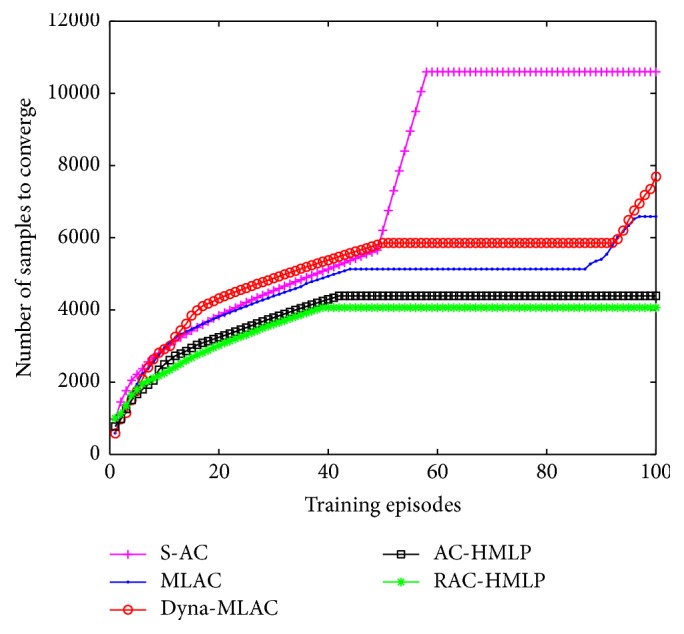
Comparisons of different algorithms in sample efficiency.

The comparisons of sample efficiency are shown in Figure 8. The required samples for S-AC, MLAC, Dyna-MLAC, AC-HMLP, and RAC-HMLP to converge are 10595, 6588, 7694, 4388, and 4062, respectively. As in pole balancing problem, RAC-HMLP also requires the least samples while S-AC needs the most to converge. The difference is that Dyna-MLAC requires samples slightly more than MLAC. The ascending curve of Dyna-MLAC at the end of the training demonstrates that it has not converged. The frequent visitation to the barrier in Dyna-MLAC leads to the quick descending of the value functions. As a result, enormous samples are required to make these value functions more prone to the true ones.

**Figure 9:**
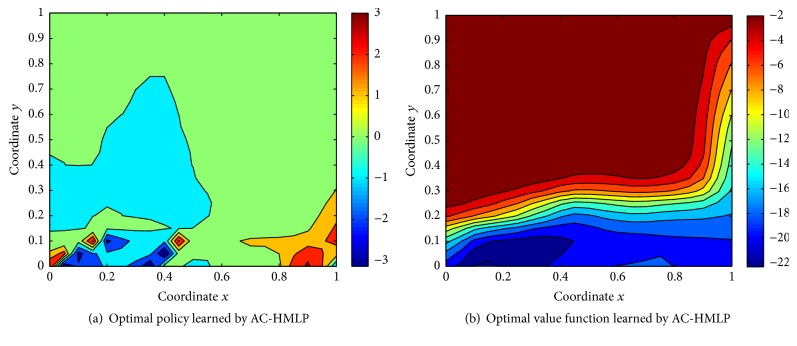
Final optimal policy and value function after training.

After the training ends, the approximate optimal policy and value function are obtained, shown in Figures 9(a) and 9(b). It is noticeable that the low part of the figure is explored thoroughly, so that the policy and the value function are very distinctive in this region. For example, in most of the region in Figure 9(a), a larger angle is needed for the agent so as to leave the current state. Clearly, the nearer the current state and the low part of Figure 9(b), the smaller the corresponding value function. The top part of the figures may be not frequently or even not visited by the agent, resulting in the similar value functions. The agent is able to reach the goal only with a gentle angle in these areas.

## 6. Conclusion and Discussion

This paper proposes two novel actor-critic algorithms, AC-HMLP and RAC-HMLP. Both of them take LLR and LFA to represent the local model and global model, respectively. It has been shown that our new methods are able to learn the optimal value function and the optimal policy. In the pole balancing and continuous maze problems, RAC-HMLP and AC-HMLP are compared with three representative methods. The results show that RAC-HMLP and AC-HMLP not only converge fastest but also have the best sample efficiency.

RAC-HMLP performs slightly better than AC-HMLP in convergence rate and sample efficiency. By introducing *ℓ*_2_-regularization, the parameters learned by RAC-HMLP will be smaller and more uniform than those of AC-HMLP, so that the overfitting can be avoided effectively. Though RAC-HMLP behaves better, its improvement over AC-HMLP is not significant. Because AC-HMLP normalizes all the features to [0,1], the parameters will not change heavily. As a result, the overfitting can also be prohibited to a certain extent.

S-AC is the only algorithm without model learning. The poorest performance of S-AC demonstrates that combining model learning and AC can really improve the performance. Dyna-MLAC learns a model via LLR for local planning and policy updating. However, Dyna-MLAC directly utilizes the model before making sure whether it is accurate; additionally, it does not utilize the global information about the samples. Therefore, it behaves poorer than AC-HMLP and RAC-HMLP. MLAC also approximates a local model via LLR as Dyna-MLAC does, but it only takes the model to update the policy gradient, surprisingly with a slightly better performance than Dyna-MLAC.

Dyna-MLAC and MLAC approximate the value function, the policy, and the model through LLR. In the LLR approach, the samples collected in the interaction with the environment have to be stored in the memory. Through KNN or *K*-d tree, only *L*-nearest samples in the memory are selected to learn the LLR. Such a learning process takes a lot of computation and memory costs. In AC-HMLP and RAC-HMLP, there is only a parameter vector to be stored and learned for any of the value function, the policy, and the global model. Therefore, AC-HMLP and RAC-HMLP outperform Dyna-MLAC and MLAC also in computation and memory costs.

The planning times for the local model and the global model have to be determined according the experimental performance. Thus, we have to set the planning times according to the different domains. To address this problem, our future work will consider how to determine the planning times adaptively according to the different domains. Moreover, with the development of the deep learning [33, 34], deep RL has succeeded in many applications such as AlphaGo and Atari 2600 by taking the visual images or videos as input. However, how to accelerate the learning process in deep RL through model learning is still an open question. The future work will endeavor to combine deep RL with hierarchical model learning and planning to solve the real-world problems.

## Figures and Tables

**Algorithm 1:**
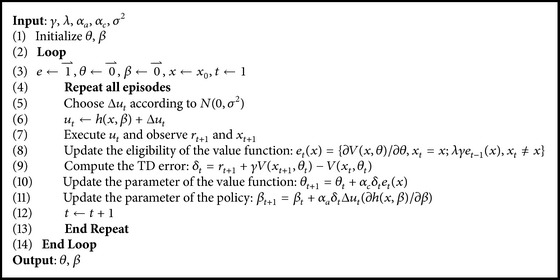
S-AC.

**Algorithm 2:**
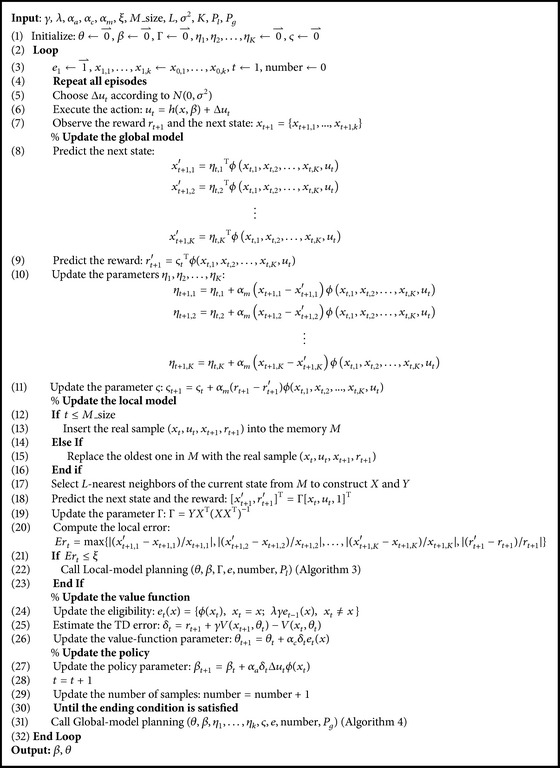
AC-HMLP algorithm.

**Algorithm 3:**
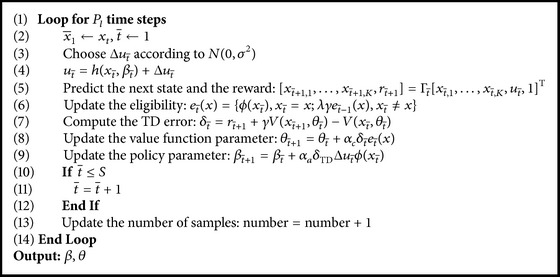
Local model planning (*θ*, *β*, Γ, *e*, number, *P*_*l*_).

**Algorithm 4:**
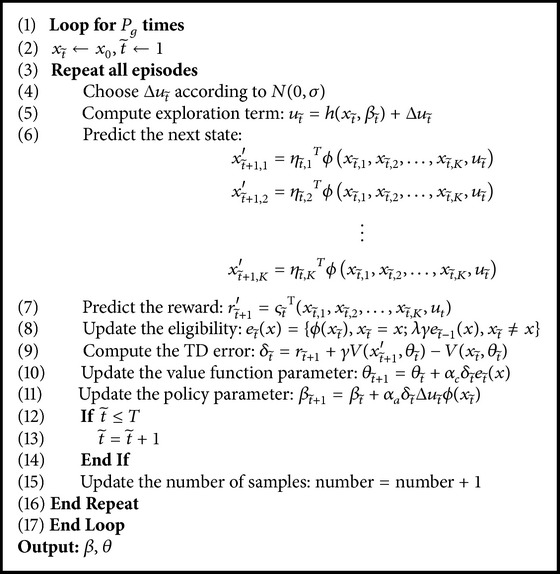
Global model planning (*θ*, *β*, *η*_1_,…, *η*_*k*_, *ς*, *e*, number, *P*_*g*_).

**Table 1:** Parameters settings of RAC-HMLP and AC-HMLP.

Parameter	Symbol	Value
Time step	*T*_*s*_	0.1
Discount factor	*γ*	0.9
Trace-decay rate	*λ*	0.9
Exploration variance	σ^2^	1
Learning rate of the actor	*α*_*a*_	0.5
Learning rate of the critic	*α*_*c*_	0.4
Learning rate of the model	*α*_*m*_	0.5
Error threshold	*ξ*	0.15
Capacity of the memory	*M*_size	100
Number of the nearest samples	*L*	9
Local planning times	*P*_*l*_	30
Global planning times	*P*_*g*_	300
Number of components of the state	*K*	2
Regularization parameter of the model	*ℓ*_*m*_	0.2
Regularization parameter of the critic	*ℓ*_*c*_	0.01
Regularization parameter of the actor	*ℓ*_*a*_	0.001
